# Reproducibility of Bioelectrical Impedance Analysis in Pregnancy and the Association of Body Composition with the Risk of Gestational Diabetes: A Substudy of MUMS Cohort

**DOI:** 10.1155/2020/3128767

**Published:** 2020-09-22

**Authors:** Michelle Bai, Daniella Susic, Anthony J. O'Sullivan, Amanda Henry

**Affiliations:** ^1^School of Women's and Children's Health, UNSW Medicine, Sydney, Australia; ^2^Department of Women's and Children's Health, St. George Hospital, Sydney, Australia; ^3^Department of Endocrinology, St. George Hospital, Sydney, Australia; ^4^St. George and Sutherland Clinical School, UNSW Medicine, Sydney, Australia; ^5^The George Institute for Global Health, UNSW Medicine, Sydney, Australia

## Abstract

**Introduction:**

Bioelectrical impedance analysis (BIA) is a rapid and noninvasive method of body composition analysis; however, reproducibility between BIA instruments in pregnancy is uncertain. Adverse maternal body composition has been linked to pregnancy complications including gestational diabetes mellitus (GDM). This study aimed to evaluate the reproducibility of three BIA instruments in pregnancy and analyse the relationship between the body composition and the GDM risk.

**Methods:**

A prospective cohort (*n* = 117) of women with singleton pregnancies participating in the Microbiome Understanding in Maternity Study (MUMS) at St. George Hospital, Sydney, Australia. Anthropometric measurements and BIA body composition were measured at ≤13 weeks (T1), 20–24 weeks (T2), and 32–36 weeks (T3) of gestation. Body fat percentage (BFP), total body water (TBW), and impedance were estimated by three BIA instruments: Bodystat 1500, RJL Quantum III, and Tanita BC-587. GDM status was recorded after 75 g oral glucose tolerance test was performed at 28 weeks or earlier. Agreement between BIA instruments was assessed using Bland–Altman analysis. Logistic regression modelling explored associations of BFP with GDM.

**Results:**

Method comparison reproducibility between Bodystat and RJL was stronger than between Bodystat and Tanita for both BFP and TBW% at all three time points. RJL overestimated BFP on average by 3.3% (*p* < 0.001), with limits of agreement within ±5% for all trimesters. Average BFP was not significantly different between Tanita and Bodystat although limits of agreement exceeded ±5%. GDM diagnosis was independently associated with increased BFP in T1 (adjusted OR 1.117 per 1% increase; 95% CI 1.020–1.224; *p*=0.017) and in T2 (adjusted OR 1.113 per 1% increase; 95% CI 1.010–1.226; *p*=0.031) and with Asian ethnicity in all models (OR 7.4–8.1).

**Conclusion:**

Reproducibility amongst instruments was moderate; therefore, interchangeability between instruments, particularly for research purposes, cannot be assumed. In this cohort, GDM risk was modestly associated with increasing BFP and strongly associated with Asian ethnicity.

## 1. Introduction

Pregnancy is a state involving many physical changes, including the developing fetoplacental unit, amniotic fluid, breast and uterine tissue, body water, and maternal fat [1]. One of the four-compartment models of body composition consists of fat mass (FM) and fat-free mass (FFM), which is further divided into total body water (TBW), proteins, and minerals [2]. Examining shifts in maternal body composition may better inform our understanding of physiological and pathological processes in pregnancy. Commonly used adiposity measures such as gestational weight gain and body mass index (BMI) are only surrogate markers of adiposity and are therefore inadequate when assessing high-risk pregnancies [3].

The clinical utility of body composition measurement in pregnancy is an ongoing area of research. Body fat percentage (BFP), calculated as FM divided by total body mass multiplied by 100%, increases significantly during pregnancy [4]. Increased BFP during pregnancy has been associated with an increased risk of pregnancy complications such as gestational diabetes mellitus (GDM) [5–8] and hypertensive disorders of pregnancy [9, 10]. BFP has been suggested to be a better predictor of GDM than BMI (*r* = 0.68 vs. 0.57, *p* < 0.01) [7]. However, this correlation has only been studied in Asian women and may not apply in other ethnicities with different risk profiles and prevalence of GDM.

Physiological TBW increase in pregnancy is attributed to plasma volume expansion, amniotic fluid, and water in the fetus, placenta, and reproductive organs. TBW is associated with the development of preeclampsia [10, 11] and infant birth weight [12, 13]. This may be related to haemodynamic adaptations to pregnancy, particularly in the first and second trimesters, causing a shift in the volume distribution and alterations in the cardiovascular system regarding capillary permeability and shifts in the haemodynamic state [10].

An emerging area of research explores the role of the human microbiome, that is, the population of microbes occupying various body sites, in mediating the complex physiological changes of pregnancy [14, 15]. Studies in both pregnant and nonpregnant populations suggest a link between the microbiome, energy storage, and metabolism [16–18]. Further research is needed to examine the relationship between the microbiome and body fat accumulation in pregnancy.

Measuring body composition in pregnancy is challenging due to unique pregnancy-related changes in physiology and methodological restrictions. A recent review discussed the advantages and disadvantages of various methods currently available for body composition analysis in pregnancy [19]. Bioelectrical impedance analysis (BIA) is a noninvasive, rapid, inexpensive, and portable method which is safe to use in pregnancy. Single-frequency BIA instruments pass a 50 kHz alternating current through the body via electrodes, and TBW is calculated from the total impedance of electrical flow by the tissues [20]. BFP and FFM are then estimated using algorithms based on population data.

Conventional BIA systems operate using a hand-to-foot configuration, while newer foot-to-foot systems have been developed which utilise a scale configuration with foot-pad electrodes. These scales measure standing impedance and body weight measurements simultaneously, offering greater ease and speed of measurement [21]. BIA scales have been validated and shown to be comparable to conventional arm-to-leg BIA [21, 22]. However, these studies were supported by the manufacturer, thus potentially subject to bias, and were not performed in pregnancy.

Current proprietary, population-based algorithms utilised by commercial BIA instruments may not be valid in pregnancy since inherent assumptions regarding density and composition of FFM may be violated [23]. At present, there have been no published studies analysing method comparison reproducibility of different BIA instruments in pregnancy. There have also been no studies comparing foot-to-foot with hand-to-foot BIA configurations in pregnancy. Sources of error between instruments include, although are not limited to, technique of measurement, such as electrode positioning and skin cleanliness, raw reactance and resistance values, and preprogrammed algorithms utilised [20]. Potential differences between instruments may preclude interchangeability of measurements of individual subjects in clinical practice or research settings.

The Microbiome Understanding in Maternity Study (MUMS) is a prospective cohort study aiming to examine the relationships between the maternal and fetal microbiome, metabolic physiology, clinical history, and pregnancy and infancy outcomes. The metabolic profile of participants includes body composition measured using BIA. To ensure that findings from MUMS and other studies by our research group are clinically valid, the BIA instrument employed should be comparable to other instruments available to researchers and clinicians. There may also be a role for BIA in routine antenatal care in future if it is found to be an effective and reliable estimate of body composition in pregnancy.

The aims of this study were therefore (1) to evaluate the reproducibility of three BIA instruments used in pregnancy by comparing predicted BFP, TBW, and impedance and (2) to analyse the relationship between body composition and the development of GDM in pregnant women.

## 2. Materials and Methods

### 2.1. Subjects and Study Design

This is a substudy of the prospective longitudinal cohort, “Microbiome Understanding in Maternity Study” (MUMS). The study was conducted in pregnant women recruited from the antenatal clinic of St. George Hospital, Sydney, Australia. Women were eligible for the study if aged 18 years or over with a singleton pregnancy, ≤13 weeks of gestation at enrolment, and capable of giving informed consent. Exclusion criteria included women requiring interpreter services and/or planning to have a home birth. Participants were considered as “high risk” at the time of enrolment based on the past history of hypertension or diabetes in pregnancy, chronic hypertension, prepregnancy diabetes, and/or BMI ≥30. Gestational age was determined by the last menstrual period and confirmed by the first trimester dating ultrasound.

Consenting women were studied once during each trimester of pregnancy for the substudy: at ≤13 weeks (T1), 20–24 weeks (T2), and 32–36 weeks (T3) of gestation. At each time point, body composition, weight, and waist and hip circumferences were measured. Height was measured at T1. Demographic data and medical history were obtained from all subjects. GDM status was recorded after a routine 28-week glucose tolerance test (GTT), unless diagnosed earlier. Diagnosis was made if fasting glucose ≥5.1 mmol/L, 1 hr ≥ 10.0 mmol/L, or 2 hr ≥ 8.5 mmol/L [24]. Study data were collected and managed using REDCap electronic data capture tools hosted at the University of New South Wales [25].

### 2.2. Anthropometric Measurements

All subjects were prepared prior to testing using the following protocol: barefoot, wearing light clothing, jewellery removed, not sweating, and bladder emptied [26]. All measurements were completed by one of two investigators, according to the manufacturer instructions.

Body weight was measured in 0.1 kg increments using Tanita BC587 scales (Tanita Corp., Tokyo, Japan). Height was measured using a wall-mounted stadiometer (model 602VR, Holtain Ltd., Crymych, Wales). Waist and hip circumferences were measured using a flexible, nonstretch, fibre glass measuring tape with markings in millimetres. Waist circumference was measured at the level of the umbilicus. Hip circumference was measured around the widest circumference of the buttocks. BMI was calculated as the body weight in kilograms divided by the square of the height in metres (kg/m^2^). BMI cutoff points from the World Health Organization [27] were used to define normal (BMI 18.50–24.99 kg/m^2^), overweight (BMI 25–29.99 kg/m^2^), and obese (BMI ≥ 30 kg/m^2^).

### 2.3. Body Composition Measurements

BIA body composition was assessed using three devices:

1. Bodystat 1500 (Bodystat Ltd., Douglas, Isle of Man, United Kingdom)
2. RJL Systems Quantum III BIA analyzer (RJL Systems, Clinton Township, Michigan, USA)
3. Tanita BC-587 scales (Tanita Corp., Tokyo, Japan)

The subjects' height, age, sex, weight, frame size, and activity level were inputted to the devices. BFP (% of total body weight) and TBW (% of total body weight) were estimated by all instruments, and impedance (Ω) was measured by Bodystat and RJL and recorded. Three trials for each device were performed for the first 50 subjects, after which test-retest repeatability was verified, and thereafter, only one measurement per device was taken. Where three trials were performed, testing was completed within five minutes. Total time to complete all measurements at each visit was approximately 15 minutes.

Bodystat and RJL are both single-frequency (50 kHz) hand-to-foot BIA devices, which were calibrated using a 500 Ω resistor regularly. Four adhesive Ag/AgCl electrodes (BIA Electrodes 92500, RJL Systems, Clinton Township, Michigan, USA) were placed on the dorsal surfaces of the right hand and right foot. Prior to electrode placement, the skin of the four locations was wiped with an alcohol swab. The subjects reclined in a supine position on an examination table, with legs slightly apart and arms at an approximate 30° angle to the body. The subjects were instructed to relax and lie still whilst measurements were taken.

The Tanita instrument is a single-frequency (50 kHz) foot-to-foot BIA scale. Prior to measurement, subjects were instructed to wipe the soles of their feet with an alcohol swab. The subjects then stepped onto the scale, ensuring that the heels of both feet were positioned above the two heel electrodes. The subjects were then instructed to stand still with arms by their side until the body composition measurement was complete.

Bodystat was used as the reference method due to the previous validation in our laboratory with isotopic dilution with tritiated water in normal nonpregnant subjects [28] and with DEXA in 18 women, 3–5 days postpartum. Bodystat and DEXA correlated well for FM (kg) (*r*^2^ = 0.92, *p*=0.0001), but Bodystat overestimated FM by 2.1 kg compared to DEXA. Bodystat has been used in other pregnancy studies [4, 28–30] and has had extensive external validation with a range of reference techniques including total body water, hydrostatic weighing, DEXA, and air displacement plethysmography [31].

### 2.4. Statistical Analysis

Data were analysed using IBM SPSS version 26 for Windows (IBM Corp., Armonk, NY, USA). Statistical significance was defined as *p* < 0.05. Graphical figures were generated using GraphPad Prism version 8.00 for Mac (GraphPad Software, La Jolla, California, USA). Descriptive data are expressed as mean ± SD for normally distributed continuous data, median (IQR) for nonnormally distributed continuous data, and number (percentage) for categorical data. Cross-sectional analyses included all data available for each time point, and longitudinal analyses were performed using data from participants who had completed all three visits.

#### 2.4.1. Terminology

The analysis of clinical measurement error involves many terms which have been used with varying degrees of consistency throughout the literature. According to Bartlett and Frost, repeatability refers to “the variation in repeat measurements made on the same subject under identical conditions.” [32] For this study, the more specific term “test-retest repeatability” is used to refer to the variation in measurements made by the same BIA instrument on the same patients under the same conditions [33]. In a method comparison study, reproducibility refers to “the variation in measurements made on a subject in changing conditions.” [32] In this study, reproducibility refers to the comparison of the three BIA instruments against each other.

Repeatability and reproducibility studies can be assessed using reliability (inherent variability in the true difference between measurements) and/or agreement (the quantified variation between measurements). In this study, reliability was determined using the intraclass correlation coefficient (ICC) and agreement using Bland–Altman analysis [34].

#### 2.4.2. Test-Retest Repeatability

ICCs were computed to assess test-retest repeatability. Analysis incorporated three repeat trials at each time point by each device for BFP and TBW%. ICC estimates and their 95% confidence intervals were calculated based on a single-rating (*k* = 3), absolute-agreement, 2-way mixed-effect model. ICC values ≤ 0.5, 0.5–0.75, and 0.75–0.9 and ≥0.9 were indicative of poor, moderate, good, and excellent reliability, respectively [33].

#### 2.4.3. Method Comparison Reproducibility

ICCs were computed to assess the reliability component of reproducibility. ICC (3, 1) estimates and their 95% confidence intervals were calculated based on a single-rating (*k* = 2), consistency, 2-way mixed-effect model [33].

Bland–Altman plots were plotted to determine the agreement component of reproducibility between BIA instruments [34]. The mean of three repeated measures was calculated for each individual to evaluate reproducibility. The difference between measures and the mean of measures was calculated for Bodystat vs. RJL and Bodystat vs. Tanita as Bodystat minus comparison device. Mean of differences and ±1.96 SD limits of agreement (LOA) were plotted on scatterplots.

#### 2.4.4. Clinical Correlates of Body Composition

Longitudinal changes in body composition were assessed using one-way repeated-measures ANOVA. Greenhouse–Geisser corrections were applied where the sphericity assumption was violated. *Post hoc* pairwise comparisons were made where the main effect was significant, with Bonferroni adjustment for multiple corrections. Univariate logistic regression was used to identify potential predictors of GDM and confounders. Variables were chosen for inclusion in the multiple logistic regression models if univariate analysis was significant or if there was a known predictor from the literature.

## 3. Results

### 3.1. Study Population

During April 2018 to January 2019, 117 women were recruited and completed the T1 visit, 103 completed the T2 visit, and 98 completed the T3 visit. In total, there were 11 withdrawals from the parent study, 3 first-trimester miscarriages, 3 preterm births prior to the T3 visit, 1 lost to follow-up, and 1 moved out of the area. Participant follow-up is detailed in Supplementary Figure 1. Participant characteristics are shown in Table 1. T1 visits occurred at 11.6 ± 1.4 weeks, T2 visits at 21.8 ± 1.7 weeks, and T3 visits at 33.9 ± 1.5 weeks. In terms of BMI, 25% of participants were overweight, and 21% were obese. Of 95 women completing GDM screening, 17 (18%) were diagnosed with GDM. Five GDM women were diet-controlled, four on metformin treatment, seven on insulin treatment, and one on combined metformin and insulin treatment.

### 3.2. Method Comparison of BIA Instruments

#### 3.2.1. Test-Retest Repeatability

Excellent test-retest repeatability between three repeat measurements was shown for all three instruments. Less variation was present in the measurement of BFP (ICC range 0.993–1.000) versus TBW% (ICC range 0.988–1.000). The excellent repeatability was consistent during all three trimesters of pregnancy.

#### 3.2.2. Method Comparison Reproducibility

**Figure 1:**
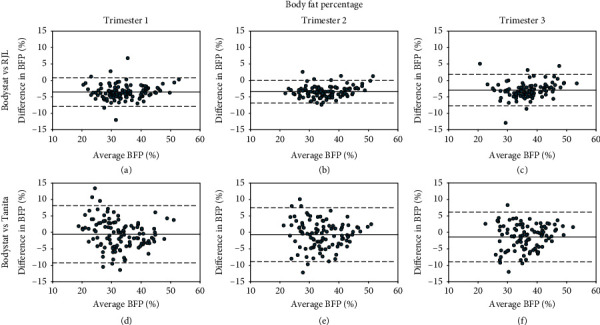
Bland–Altman plots of the differences of BFP against average BFP between two methods: (a–c) Bodystat vs. RJL and (d–f) Bodystat vs. Tanita. Differences in BFP are calculated as Bodystat minus comparison method (RJL or Tanita). BFP: body fat percentage.

**Figure 2:**
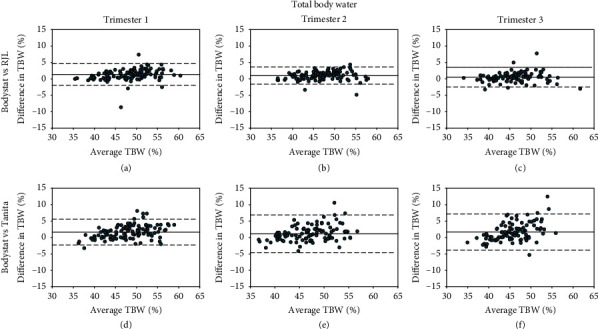
Bland–Altman plots of the differences of TBW% against average TBW% between two methods: (a–c) Bodystat vs. RJL and (d–f) Bodystat vs. Tanita. Differences in TBW% are calculated as Bodystat minus comparison method (RJL or Tanita). TBW: total body water.

**Figure 3:**
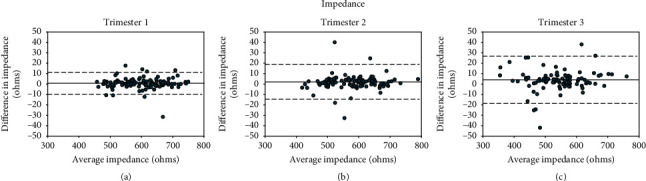
Bland–Altman plots of the differences of impedance against average impedance between two methods, Bodystat and RJL, in (a) T1, (b) T2, and (c) T3.

Tables 2–4 show the ICC and Bland–Altman average differences and LOA between the three instruments, and Bland–Altman plots are shown in Figures 1–3. Overall, RJL demonstrated better agreement with Bodystat than Tanita vs. Bodystat due to smaller LOA and stronger reliability as demonstrated by the ICC. The agreement was consistent throughout the trimesters for each method comparison. TBW% was measured in closer agreement than BFP. When the Bland–Altman analysis was performed separately for normal BMI and overweight/obese subgroups, the mean bias and LOA did not vary significantly.

*(1) Body Fat Percentage (BFP)*. The reliability of BFP between Bodystat vs. RJL was excellent (ICCs all ≥0.9) whilst Bodystat vs. Tanita was good (Table 2). RJL overestimated BFP compared with Bodystat significantly (*p* < 0.001); however, the LOA were all within clinically acceptable limits of ±5% (Figures 1(a)–1(c)). Average BFP was not significantly different between Bodystat vs. Tanita, except for in T3; however, the LOA were greater than ±5% (Figures 1(d)–1(f)).

*(2) Total Body Water*. The reliability of TBW% measurements between Bodystat vs. RJL was excellent whilst Bodystat vs. Tanita was good to excellent (Table 3). Both RJL and Tanita significantly overestimated TBW% compared with Bodystat in all trimesters (*p* < 0.001); however, the LOA for Bodystat vs. RJL were all within clinically acceptable limits of ±5% (Figures 2(a)–2(c)), whilst the LOA for Bodystat vs. Tanita were greater than ±5% for T2 and T3 (Figures 2(d)–2(f)).

*(3) Impedance*.  Table 4 shows the reproducibility analysis for the measurement of impedance by Bodystat and RJL. Reliability was excellent across the three trimesters. RJL underestimated impedance compared with Bodystat significantly in T2 (*p*=0.01) and T3 (*p* < 0.001) but not in T1. Agreement was good in T1 as LOA were approximately ±10 Ω (1.7% of mean T1 impedance). Later trimesters had poorer agreement, with T3 LOA approximately ±23 Ω (4.2% of mean T3 impedance) with several outliers.

### 3.3. Clinical Utility of Body Composition

#### 3.3.1. Body Composition Changes during Pregnancy

Longitudinal changes in anthropometric measurements and BIA body composition of the cohort are outlined in Table 5. As expected, mean BFP differed significantly across pregnancy. Mean BFP increased by 1.8% on average (*p* < 0.001) from T1 to T2 and by average 1.7% (*p* < 0.001) from T2 to T3. BFP increased on average by 3.4% (95% CI 2.4–4.5, *p* < 0.001) and FM by 5.8 kg (95% CI 5.0–6.7 kg, *p* < 0.001) from T1 to T3. Mean TBW% all differed significantly between trimesters of pregnancy, decreasing by 1.5% on average from T1 to T2 and by 1.2% from T2 to T3.

Overweight/obese subjects had significantly higher BFP, FM, and TBW (L) and lower TBW% compared to women with normal baseline BMI in all three trimesters (all *p* < 0.001). However, difference in BFP from T1 to T3 did not differ significantly between normal BMI and overweight/obese (3.4 ± 4.2% vs. 3.5 ± 4.1%, *p*=0.89).

#### 3.3.2. Prediction of GDM

Table 6 presents univariate logistic regression analysis for risk factors of GDM including BIA body composition parameters. Multiple logistic regression analyses of three models are then presented in Table 7. T1 BFP, T2 BFP, and initial BMI were significantly associated with an increased risk of GDM in models 1, 2, and 3, respectively. These three variables were analysed in separate models due to collinearity with each other. The models explained 22–24% of the total variability of the GDM status. When adjusted for initial BMI, neither T1 BFP nor T2 BFP reached statistical significance. Asian ethnicity was associated with increased GDM in all models.

## 4. Discussion

This study addressed questions regarding reproducibility of BIA instruments when applied in pregnancy, as well as the relationship between body composition and GDM in pregnancy. The results showed excellent test-retest repeatability by all instruments in all three trimesters. The method comparison found that reproducibility between instruments was variable, with agreement between Bodystat and RJL closer than between Bodystat and Tanita. Overall, the three instruments cannot be used interchangeably, although Bodystat and RJL estimates were mostly within clinically acceptable limits from one another. T1 BFP and T2 BFP were significantly associated with an increased risk of GDM in this population.

### 4.1. Test-Retest Repeatability

The test-retest repeatability demonstrated by all instruments was excellent, which is consistent with the previous studies of nonpregnant subjects [26]. The smallest range of the ICC was with Bodystat, then RJL, and widest with Tanita. This suggests that, in future protocols with repeated measures involving these instruments, a single measurement will be sufficient. This would minimise the time taken for measurements, which is especially preferred since many women find it uncomfortable to lie supine in the later stages of pregnancy. The excellent repeatability also implies that the instruments would be capable of distinguishing relatively small differences within a subject, such as in a longitudinal study design.

### 4.2. Method Comparison Reproducibility

Bodystat was used as the reference method due to the previous validation in our laboratory [29, 35]. Overall, the results showed that the three instruments cannot be used interchangeably for an individual's single or serial measurements. Consistent choice of the instrument when assessing the change in body composition of an individual or when assessing a small sample size is recommended. When compared to Bodystat, estimates of BFP and TBW% by RJL demonstrated greater reliability and narrower LOA than estimates by Tanita. The excellent agreement of TBW% between RJL and Bodystat suggests that the two instruments may be used interchangeably for studies where the outcome is TBW% rather than BFP.

Deurenberg [36] suggested that greater individual error exists in obese compared to normal weight subjects; however, this was not observed in this study. After stratifying patients based on normal BMI and overweight/obese, the reproducibility of the instruments did not change, which suggests the validity of our results for measuring patients of different BMI. Several outliers which were identified in our data may be due to the operator error, environmental electrical interference, or instrument technical error.

The finding of the close agreement of impedance between RJL and Bodystat is reassuring. LOA within 10–15 Ω infer clinically insignificant changes in BFP or TBW% estimation [36]. This implies that the variation in BFP and TBW% estimations are caused by the difference in prediction equations used by each instrument rather than an inherent difference in the bioelectrical impedance technology. The poorer agreement in T3 may be due to greater dynamic fluid shifts in later gestation when a woman is lying supine; however, it did not appear to influence the agreement within BFP and TBW% comparisons.

The comparison between Tanita and Bodystat was relatively poorer; although Tanita did not tend to overestimate or underestimate BFP or TBW% significantly, the LOA were wider than clinically acceptable. This moderate reproducibility may be attributed to the difference in the mode of measurement. The Tanita scales predominately estimate lower-limb composition since the electrodes are bipedal, whereas RJL and Bodystat are both configured hand-to-foot and hence incorporate the upper limb and abdomen in their estimations. The LOA in this study were similar to those of studies comparing foot-to-foot BIA with DEXA [37] and a four-compartment model [22]. Unexpectedly, the reproducibility of Tanita did not worsen as gestation increased, despite abdominal changes being more significant in late pregnancy. This suggests that the hand-to-foot configuration is less influenced by abdominal body geometry than presupposed. This consideration has also been raised when applying BIA on obese subjects [36] and ascites patients [38].

We believe this is the first published study to compare hand-to-foot BIA and foot-to-foot BIA in a pregnant population. Although foot-to-foot BIA demonstrated poorer reproducibility when compared to conventional hand-to-foot BIA, the agreement between Tanita and Bodystat was still moderate, and so potentially acceptable for use in large clinical studies and assessment of population means. The advantages of BIA scales are their relatively low cost, ease of use since they are performed whilst the patient is standing, and their convenience and portability.

Lukaski et al. [1] and van Raaij et al. [23] developed multiple regression equations to estimate body composition from BIA in a pregnant population. van Raaij et al. published equations for estimating FM at 10-week gestation intervals. We compared FM estimated by Bodystat algorithms with FM calculated using an equation described by Most et al. [19] which was adapted from van Raaij's equations, for any gestational age. Reliability was excellent between the two measures, with ICC greater than 0.98 for all trimesters. However, agreement assessed by Bland–Altman plots between Bodystat and published equations was poor, with Bodystat measuring FM lower by approximately 1 kg, and LOA were approximately ±2-3 kg. This is likely due to the different populations from which the equations were derived. van Raaij's equations were derived from a small sample of well-nourished pregnant Dutch women, whilst Bodystat algorithms were derived from nonpregnant subjects. It is important to note that neither methods are ideal as they may not be applicable for women with pregnancies complicated with fluid imbalance or for all ethnicities.

We were unable to use the equation for TBW in pregnancy published by Lukaski et al. due to its requirement of haematocrit measurement. Furthermore, the complexity of the equations renders them inconvenient for routine clinical use and at a risk of incorrect use. Lukaski's equations incorporate raw BIA values of reactance and resistance, which are not output values of all BIA instruments. Further development of gestation and ethnicity-specific BIA equations is necessary for the establishment of reference ranges for body composition in pregnancy.

### 4.3. Change in Body Composition during Pregnancy

With regard to the body composition change, in the present study, there was on average a 10 kg increase in body weight, 3.4% increase in BFP, and 5.8 kg increase in absolute FM. The rate of fat accumulation was similar from early to late pregnancy. These findings are consistent with a prior study performed at our unit of 26 women, which showed that BFP increased by 4.5% and absolute FM by 7.0 kg on average between the first and third trimesters [4]. However, our subjects gained more absolute body fat compared to other previous studies [39, 40]. Factors which may contribute to the inconsistencies include differences in the method of estimation, ethnicity, dietary intake, activity levels, individual variability in fat deposition, and higher proportion of overweight/obese subjects in our study. In our cohort 18% developed GDM, which is higher than the reported population incidence of 12–14% in Australia [41] and 9.8% in the USA [42]. In the other past studies (all of which were in the USA except one), either none of the subjects developed GDM [39] or GDM status was not reported [40].

### 4.4. Clinical Correlates of Maternal Body Composition and GDM

In the present study, we found that increased BFP in the first two trimesters of pregnancy is associated with the development of GDM. BFP as a predictor of GDM has also been shown by previous studies performed on solely Asian populations [5–8], and the odds ratios in the present study are similar to those prior studies.

Interestingly, in subjects who had normal initial BMI and GDM still had (nonsignificantly) higher BFP in the first two trimesters compared to those of normal BMI and no GDM. Gómez‐Ambrosi et al. [43] found BPF was higher in normal BMI women with prediabetes or type 2 diabetes compared to those with normoglycaemia. Screening BFP in pregnancy may provide clinicians additive information to assess GDM risk, particularly in cases where BMI does not correlate well with body fat, such as high muscle mass, significant fluid retention, and hidden adiposity (low BMI with high BFP). Further investigation into the role of BFP in GDM progression is required with a larger, sufficiently powered sample size in a multiethnic or non-Asian population.

There is a current interest surrounding the link between body fat and GDM since body fat has been related to insulin resistance, increased risk of type 2 diabetes mellitus, and HbA1c levels in diabetic patients [43, 44]. Dysbiosis of gut microbiota has been hypothesised as a mediator between adiposity and metabolic changes of pregnancy. A study by Koren et al. [14] showed that GDM-positive women tended to have less microbial richness at T1 compared to GDM-negative. In the same study, faecal transplantation was used to administer gut microbiota from pregnant women in first and third trimesters to germ-free mice. Greater adiposity, insulin insensitivity, and inflammatory responses, resembling the metabolic syndrome, were induced in the third-trimester versus first-trimester mice, suggesting there is a microbial component mediating host immunity and metabolism. High maternal BMI is associated with aberrant gut microbiota, with *Bacteroides* and *S. aureus* present in significantly higher numbers in overweight pregnant women versus those of normal weight [45]. Prepregnancy weight and gestational weight gain have also been associated with differences in the gut microbiome between pregnant women [46]. Utilising BIA technology may provide a clearer understanding of the relationship between adiposity and aberrant gut microbiota as it is a more direct estimation than BMI or gestational weight gain.

Research in nonpregnant obese women suggests a microbial component to obesity and “obesogenic” gut microbiome mediated by diet [47]. Proposed mechanisms include (1) an increased capacity to harvest energy from the diet facilitated by hydrolysis of indigestible polysaccharides and subsequent rapid absorption of glucose and short-chain fatty acids and (2) host-microbial interactions that alter metabolic pathways [17, 18]. No study to date has investigated the direct relationship between maternal body composition, adiposity, and the microbiome in the context of GDM. The data of this current study, combined with the data of the ongoing MUMS, give further insight into these complex interrelationships.

### 4.5. Limitations

The main limitation of our study was the lack of a gold standard reference for body composition. Although Bodystat has previously been validated by our laboratory, there are no studies validating RJL or Tanita in pregnancy against reference methods. The use of the DEXA model is unacceptable in pregnancy due to radiation exposure. Moreover, inter- and intramanufacturer differences in DEXA have raised concern regarding validity [48]. Underwater weighing would not be tolerated by pregnant women, and CT scanning in pregnancy would be unethical for research studies. All body composition measurement methods are limited by their inability to distinguish between the mother and the fetus during pregnancy [49].

Furthermore, there was a possible influence of the adhesive electrodes on the results of method comparison reproducibility for hand-to-foot BIA. It has been shown that the choice of electrodes can significantly displace bioimpedance vector positions [50]. We used the same RJL branded electrodes for measurements by both Bodystat and RJL instruments, although Bodystat does recommend the use of their own electrodes. Another limitation was that the study was conducted at a single metropolitan site. We were unable to control for confounding factors regarding dietary habits and activity levels in the scope of this study, which may account for some of the variability in data.

## 5. Conclusion

BIA is a noninvasive, rapid, and portable method of body composition estimation, which therefore represents an ideal method for use in pregnancy in large studies and clinical settings. Bodystat, RJL, and Tanita are not interchangeable methods for individual assessment; however, they may be acceptable for assessment of large cohorts. During pregnancy, significant alterations to body composition occur, involving increased fat deposition and decrease in the body water proportion. Further studies are required to better study associations between increased body fat and development of GDM.

## Figures and Tables

**Table 1:** Subject demographic characteristics.

Characteristic	*n* = 117
Age (years)	33.1 ± 4.9
Height (cm)^*∗*^	162.8 ± 6.4
BMI (kg/m^2^)^*∗*^	25.8 ± 5.2
BMI category^*∗*^	
Underweight	1 (1%)
Normal	62 (53%)
Overweight	29 (25%)
Obese	25 (21%)
Parity	
Primiparous	46 (39%)
Multiparous	71 (61%)
High risk	47 (40.2%)
Ethnicity	
Caucasian	67 (57%)
Asian	29 (25%)
Middle Eastern	11 (9%)
Others	10 (9%)

Values are presented as mean ± SD or *n* (%). ^*∗*^Measurement performed at trimester 1. BMI: body mass index.

**Table 2:** Reproducibility in the method comparison of Bodystat, RJL, and Tanita for estimation of BFP.

	ICC^a^	ICC 95% CI	Average difference^b^	LOA	*p* value^c^
Bodystat vs. RJL					
T1	0.948	0.925–0.963	−3.52	−7.85, 0.80	<0.001
T2	0.963	0.946–0.975	−3.41	−6.88, 0.06	<0.001
T3	0.926	0.891–0.950	−2.93	−7.72, 1.87	<0.001

Bodystat vs. Tanita					
T1	0.817	0.746–0.870	−0.57	−9.28, 8.15	0.18
T2	0.810	0.732–0.868	−0.67	−8.84, 7.50	0.11
T3	0.818	0.740–0.874	−0.14	−8.99, 6.14	<0.001

^a^ *p* value for ICC all <0.001. ^b^Differences calculated as Bodystat minus RJL or Tanita. ^c^*p* value for paired *t*-test of differences between instruments. CI: confidence interval; ICC: intraclass correlation coefficient; LOA: limits of agreement; and T1/2/3: trimester 1/2/3.

**Table 3:** Reproducibility in the method comparison of Bodystat, RJL, and Tanita for estimation of TBW%.

	ICC^a^	ICC 95% CI	Average difference^b^	LOA	*p* value^c^
Bodystat vs. RJL					
T1	0.951	0.929–0.965	1.34	−1.96, 4.64	<0.001
T2	0.965	0.949–0.976	1.01	−.61, 3.63	<0.001
T3	0.951	0.927–0.967	0.55	−2.46, 3.56	<0.001

Bodystat vs. Tanita					
T1	0.926	0.895–0.949	1.66	−2.28, 5.60	<0.001
T2	0.824	0.750–0.877	1.16	−4.60, 6.91	<0.001
T3	0.805	0.723–0.865	1.67	−3.83, 7.17	<0.001

^a^ *p* value for ICC all <0.001. ^b^Differences calculated as Bodystat minus RJL or Tanita. ^c^*p* value for paired *t*-test of differences between instruments. CI: confidence interval; ICC: intraclass correlation coefficient; LOA: limits of agreement; and T1/2/3: trimester 1/2/3.

**Table 4:** Reproducibility in the method comparison of Bodystat vs. RJL for measurement of impedance (Ω).

	ICC^a^	ICC 95% CI	Average difference^b^	LOA	*p* value^c^
Bodystat vs. RJL					
T1	0.966	0.951–0.976	0.71	−9.83, 11.25	0.16
T2	0.994	0.991–0.996	2.14	−14.62, 18.90	0.01
T3	0.966	0.950–0.977	4.16	−18.54, 26.86	<0.001

^a^ *p* value for ICC all <0.001. ^b^Differences calculated as Bodystat minus RJL. ^c^*p* value for paired *t*-test of differences between instruments. CI: confidence interval; ICC: intraclass correlation coefficient; LOA: limits of agreement; and T1/2/3: trimester 1/2/3.

**Table 5:** Changes in anthropometry and BIA body composition throughout pregnancy (*n* = 98).

	Pregnancy trimesters		
	T1	T2	T3
Weight (kg)	67.6 ± 15.1	71.8 ± 15.1^a^	77.6 ± 15.5^a,b^
BMI (kg/m^2^)	25.6 ± 5.2	27.1 ± 5.2^a^	29.3 ± 5.3^a,b^
Waist circumference (cm)	86.6 ± 10.4	96.0 ± 9.6^a^	105.9 ± 9.2^a,b^
Hip circumference (cm)	102.8 ± 11.3	105.1 ± 10.7^a^	107.5 ± 10.7^a,b^
BFP (%)	32.2 ± 6.9	34.0 ± 6.8^a^	35.6 ± 6.7^a,b^
FM (kg)	22.6 ± 10.1	25.2 ± 10.2^a^	28.5 ± 10.7^a,b^
TBW (%)	49.2 ± 5.6	47.7 ± 5.2^a^	46.5 ± 5.2^a,c^
TBW (L)	32.6 ± 4.0	33.7 ± 4.2^a^	35.6 ± 4.5^a,b^

Values are reported as mean ± SD. One-way repeated-measures ANOVA is used to assess time effect, with *post hoc* pairwise analysis with Bonferroni corrections. ^a^Significantly different from the T1 value (*p* < 0.001). ^b^Significantly different from the T2 value (*p* < 0.001). ^c^Significantly different from the T1 value (*p*=0.001). BFP: body fat percentage; BIA: bioelectrical impedance analysis; BMI: body mass index; FM: fat mass; T1/2/3: trimester 1/2/3; and TBW: total body water.

**Table 6:** Univariate logistic regression analysis for the association between BIA body composition and GDM.

	OR (95% CI)	Nagelkerke *R*^2^	*p* value
Age (years)	1.007 (0.896–1.131)	0.000	0.907
Parity ≥ 1	1.184 (0.3.97–3.530)	0.002	0.762
T1 weight (kg)	1.023 (0.991–1.056)	0.031	0.168
Initial BMI (kg/m^2^)	1.820 (0.987–1.187)	0.046	0.094
T1 BFP (%)	1.073 (0.997–1.155)	0.060	0.061
T2 BFP (%)	1.074 (0.993–1.161)	0.055	0.074
BFP change from T1 to T2 (%)	0.970 (0.817–1.152)	0.002	0.731

Values are reported as mean ± SD. ^a^*p* values reported for univariate logistic regression. BFP: body fat percentage; BIA: bioelectrical impedance analysis; BMI: body mass index; GDM: gestational diabetes mellitus; and T1/2/3: trimester 1/2/3.

**Table 7:** Logistic regression models of predictors of GDM.

	Model 1		Model 2		Model 3	
	Adjusted OR (95% CI)	*p* value	Adjusted OR (95% CI)	*p* value	Adjusted OR (95% CI)	*p* value
Maternal age (years)^a^	0.960 (0.828–1.115)	0.595	0.970 (0.837–1.123)	0.684	0.993 (0.856–1.152)	0.928
Family history of diabetes	0.933 (0.271–3.206)	0.912	1.060 (0.306–3.668)	0.927	0.900 (0.259–3.130)	0.869
Asian ethnicity	7.451 (2.084–26.639)	0.002	7.436 (2.132–25.937)	0.002	8.105 (2.221–29.572)	0.002
Parity ≥ 1	1.531 (0.431–5.444)	0.510	1.848 (0.501–6.817)	0.356	1.639 (0.458–5.866)	1.639
T1 BFP (%)^a^	1.117 (1.020–1.224)	0.017	—	—	—	—
T2 BFP (%)^a^	—	—	1.113 (1.010–1.226)	0.031	—	—
Initial BMI^a^	—	—	—	—	1.140 (1.019–1.275)	0.022
Nagelkerke *R*^2^	0.236		0.237		0.227	

^a^Adjusted OR for maternal age, T1 BFP, change in BFP, and initial BMI are for one unit increase. BFP: body fat percentage; BMI: body mass index; GDM: gestational diabetes mellitus; OR: odds ratio; and T1/2/3: trimester 1/2/3.

## Data Availability

The datasets generated and/or analysed during the current study are not publicly available as results from the wider dataset of the MUMS, of which the current research is a substudy, are not yet reported.

## Abbreviations

95% CI: 95% confidence interval
BFP: Body fat percentage
BIA: Bioelectrical impedance analysis
BMI: Body mass index
DEXA: Dual-energy X-ray absorptiometry
FFM: Fat-free mass
FM: Fat mass
GDM: Gestational diabetes mellitus
ICC: Intraclass correlation coefficient
LOA: Limits of agreement
MUMS: Microbiome Understanding in Maternity Study
SD: Standard deviation
T1/2/3: Trimester 1/2/3
TBW: Total body water.

## Supplementary Materials

Supplementary Materials Figure 1: study participant flowchart.
