# Supplementary figures and images for: Reproducibility of Bioelectrical Impedance Analysis in Pregnancy and the Association of Body Composition with the Risk of Gestational Diabetes: A Substudy of MUMS Cohort
Source: J Obes. 2020 Sep 22;2020:3128767. doi: 10.1155/2020/3128767 (PMC7528004; doi:10.1155/2020/3128767)

Supplementary Figure 1:

Study Participant Flowchart

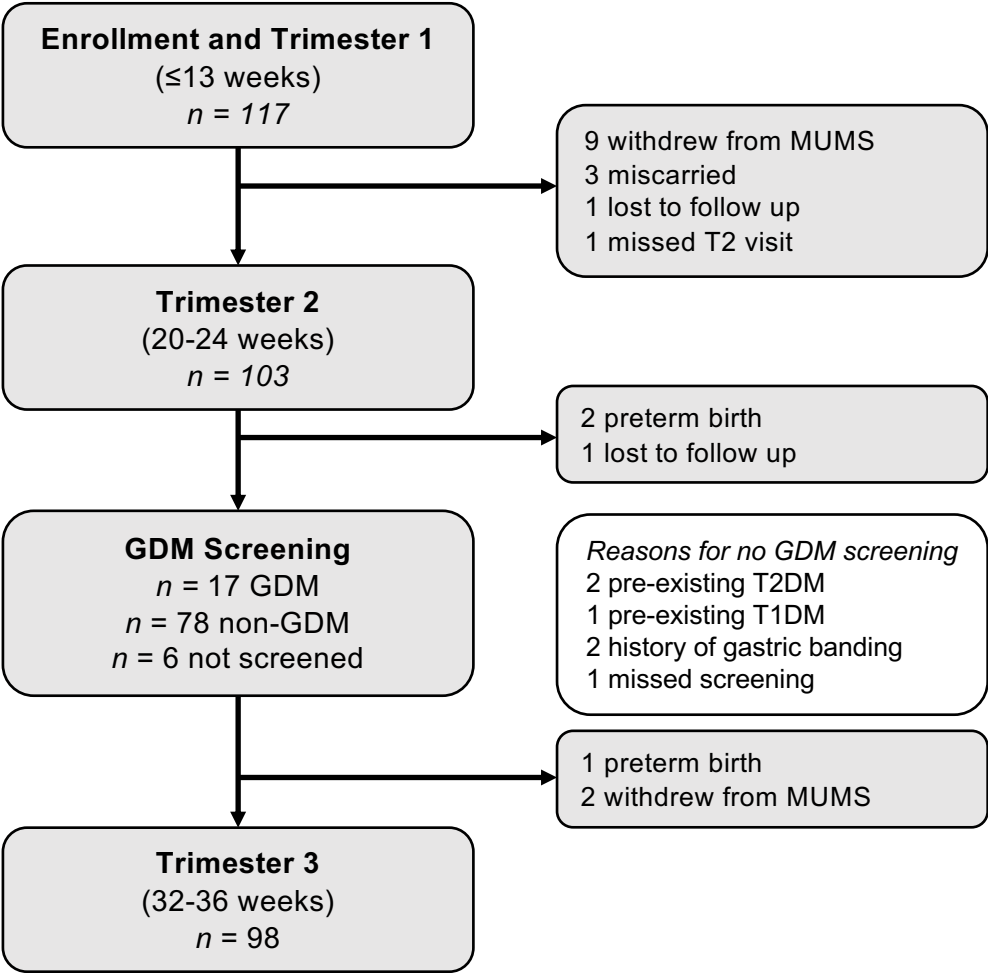

Supplement: Supplementary Materials — Figure 1: study participant flowchart. [file 3128767.f1.pdf]
